# Early developed ASD (adjacent segmental disease) in patients after surgical treatment of the spine due to cancer metastases

**DOI:** 10.1186/s13018-017-0574-3

**Published:** 2017-05-12

**Authors:** Grzegorz Guzik

**Affiliations:** Orthopedic Oncology Department, Specialist Hospital in Brzozów-Podkarpacki Oncology Center, ul Bielawskiego 18, 36-200 Brzozów, Poland

**Keywords:** Metastases, Spinal tumors, Surgical treatment of the spine, Spinal tumor resections, Spine stabilizations

## Abstract

**Background:**

The causes of ASD are still relatively unknown. Correlation between clinical status of patients and radiological MRI findings is of primary importance. The radiological classifications proposed by Pfirmann and Oner are most commonly used to assess intradiscal degenerative changes.

The aim of the study was to assess the influence of the extension of spine fixation on the risk of developing ASD in a short time after surgery.

**Methods:**

A total of 332 patients with spinal tumors were treated in our hospital between 2010 and 2013. Of these patients, 287 underwent surgeries. A follow-up MRI examination was performed 12 months after surgical treatment. The study population comprised of 194 patients. Among metastases, breast cancer was predominant (29%); neurological deficits were detected in 76 patients. Metastases were seen in the thoracic (45%) and lumbar (30%) spine; in 25% of cases, they were of multisegmental character. Pathological fractures concerned 88% of the patients. Statistical calculations were made using the *χ*2 test. Statistical analysis was done using the Statistica v. 10 software. A *p* value <0.05 was accepted as statistically significant. The study population was divided on seven groups according to applied treatment.

**Results:**

Clinical signs of ASD were noted in only seven patients. Two patients had symptoms of nerve root irritation in the lumbar spine. Twenty-two patients (11%) were diagnosed with ASD according to the MRI classifications by Oner, Rijt, and Ramos, while the more sensitive Pfirmann classification allowed to detect the disease in 46 patients (24%). Healthy or almost healthy discs of Oner type I correlated with the criteria of Pfirmann types II and III. The percentage of the incidence of ASD diagnosed 1 year after the surgery using the Pfirmann classifications was significantly higher than diagnosed according to the clinical examination.

**Conclusions:**

The incidence of ASD in patients after spine surgeries due to cancer metastases does not differ between the study groups. ASD detectability based on clinical signs is significantly lower than ASD detectability based on MR images according to the system by Pfirrmann et.al. ASD risk increase among patients with multilevel fixation.

## Background

Adjacent segmental disease following surgery of the spine represents a serious diagnostic and therapeutic problem. There are many factors causing intervertebral disc degeneration or its accelerated development. The ones that are mentioned as most important are age, location, natural aging processes, increased mobility of the segments bordering with the fusion site, changes in the forces imposed on the endplates and in their springiness, changes in intradiscal pressure, and others. It is of essential importance whether symptoms of disc and endplate degeneration occurred before the surgical treatment. What is also relevant is the level and extent of spine stabilization, treatment method and how much time elapsed since surgery. One of the most significant causes of disc degeneration is sagittal imbalances [1–4].

Diagnostics of adjacent segmental disease is particularly difficult. It should be stressed that only 25% of radiologically diagnosed abnormalities are associated with clinical signs of the disease [5–9]. The systems of classification proposed by Oner, Rijt, and Ramos and Pfirmann are of the most frequently used [8, 9]. The classification result should be compared with the clinical condition of a patient. The understanding of risk factors associated with adjacent segmental disease, and its accelerated development is of key importance for optimizing methods of the surgical treatment of spinal diseases and improving its outcomes [10–13].

The aim of the study was to assess influence of extension and type of spine stabilizations on the risk of adjacent segmental disease (ASD) in a short time after surgery. We also try to determine the correlation between the clinical and radiological symptoms of ASD and the incidence of different types of ASD in two classifications based on MRI. The analysis includes MRI and clinical findings in surgically treated patients with metastatic tumors.

## Methods

A total of 332 patients with spinal tumors were treated in our hospital between 2010 and 2013. Of these patients, 287 underwent surgeries. A retrospective examination of the patients operated on the lumbar and/or the thoracic section of the spine was carried out. The patients that underwent surgeries of the cervical spine and the patients in whom only vertebral biopsy was performed were excluded from the study. The study population comprised of a total of 247 operated patients.

The majority of the treated patients were women, representing 64% of the study population. The mean age was 63 for women and 68 for men.

Among the metastases, breast cancer dominated (29%), followed by prostate cancer (7%), multiple myeloma (12%), lung cancer (9%), kidney cancer (6%), lymphoma (3%), thyroid cancer (3%), cancer of unknown primary site (14%), and others (17%).

Neurological deficits were detected in 76 patients. Complete paralysis of the limbs, classified as Frankel A, was found in nine patients. Acute pareses diagnosed in 15 patients were classified as Frankel B, while in 26 patients as Frankel C. Minor pareses (Frankel grade D) occurred in 26 patients. There were no quadriplegic patients in our series.

In 45% of the patients, the metastases were located in the thoracic spine, while in 30% of the patients the lumbar region was involved. The patients with involvement of more than one segment of the spine accounted for 25% of cases.

The posterior elements of the spine were mainly affected, being involved in 63% of the patients. Both posterior and anterior elements were involved in 32% of the patients, and the posterior elements alone were affected in 5% of the patients.

Pathological fractures were diagnosed in 80% of the patients; while in 20% of them, the metastases did not result in fractures. Spinal instability, assessed based on the Kostiuk and Taneichi scale, was diagnosed in 64% of the patients.

Each patient had classical radiograms, CT, and MRI scans of the spine, and the following characteristics were evaluated: type, location and extent of the pathological lesions, spinal axis disorder, shape and type of fractures, and dislocation and stability of spinal segments.

The patients were divided into the following groups depending on the type of surgery: group A—patients after posterior stabilization of the spine, group B—posterior stabilization combined with laminectomy, group C—spinal laminectomy, group D—resection of the vertebral bodies through posterior approach combined with implantation of a vertebral body prosthesis and posterior stabilization, group E—resection of the vertebral body through anterior approach combined with implantation of a vertebral body prosthesis, group F—implantation of a vertebral body prosthesis with posterior stabilization, group G—anterior approach with vertebral body prosthesis and posterior stabilization. Only titanium implants were used, which allowed for the performance of subsequent CT and MRI examinations.

Due to limited survival of the patients, follow-up was 12 months after the surgery. Final analysis involved 194 patients. The rest of the patients were treated outside our center or did not respond to physician’s examination call (Table 1).

**Table 1:** Number of operated patients in different groups and their participation in a follow-up MRI performed 12 months after the surgery

Group	Group A	Group B	Group C	Group D	Group E	Group F	Group G
Number of operated patients (247)	36	118	7	16	9	38	23
Number of patients who had a follow-up MRI (194)	31	87	6	12	9	31	18

Four-level spine stabilization was the shortest one and concerned 12 patients. Five-level stabilization was done in 21 patients, sixe-level in 52 patients, seven-level in 62 patients, eight-level in 24 patients, and nine-level in 23 patients.

MRI and clinical examinations were performed to detect signs of damage to the discs in the segments adjacent to the surgically treated section of the spine. Attention was paid to increased pain intensity, its location, pain on palpation, and the range of spinal mobility. The shape of the spine, spinal axis disorders with special attention given to the sagital axis, and distortions of the natural curvatures of the spine were evaluated. What was also considered was the shape and morphology of the endplates adjacent to the stabilization site as well as disc degeneration classified using the systems by Oner, Rijt, and Ramos and Pfirmann.

The categorical variables were expressed as percentages. The inter-group differences were tested using the *χ*2 test. All statistical analyses were performed by using Statistica 10. A value of *P* < 0.05 was considered statistically significant.

The research has been performed in accordance with the declaration of Helsinki. As this retrospective analysis consists of anonymised clinical routine data, the Research Ethics Committee deems the application for and issue of an ethics approval not necessary. All the patients gave a written consent to the use of data for research, name of ethics committee: Ethics Committee in Cracov, ul Krupnicza 11a 31-123 Cracov, tel +48126191712, fax +48124225755.

## Results

A total of 194 patients had a follow-up MRI of the spine. Only seven patients in our material presented clinical signs of ASD. The patients reported chronic spinal pain. Clinical examination revealed pain on palpation of the spinous processes adjacent to the stabilized segment of the spine. Signs of nerve root irritation in the lumbar section occurred in two patients.

ASD was diagnosed in 22 (11%) patients based on the classification systems by Oner, Rijt, and Ramos. The table below demonstrates the incidence of different types of the characteristic of ASD degenerative changes classified according to the beforementioned systems (Table 2).

**Table 2:** Different types of radiologically detected (MRI) adjacent segment degenerative changes classified according to the systems by Oner, Rijt, and Ramos in relation to surgical options in the treatment of 194 follow-up patients

Group	Group A	Group B	Group C	Group D	Group E	Group F	Group G
Number of patients *N*(%)	31(16)	87(45)	6(3)	12(6)	9(5)	31(16)	18(9)
Type 1	28	76	6	9	8	28	17
Type 2	1	3	–	1	–	1	1
Type 3	1	3	–	1	–	–	–
Type 4	–	1	–	–	–	–	–
Type 5	–	–	–	1	–	1	–
Type 6	1	4	–	–	1	1	–

The incidence of various types of disc degeneration according to the Pfirmann and Metzdorf classification is presented in Table 3. It must be pointed out that following this system may entail difficulties in precise classification of some of the pathologic changes. Oner III and IV types were excluded from the Pfirmann classification and defined differently. Oner type I included healthy or almost healthy discs, of which some were classified as type II and III by Pfirmann. This resulted in an increased detectability of ASD up to a level of 46 cases (24%).

**Table 3:** Different types of radiologically detected (MRI) adjacent segment degenerative changes classified according to the Pfirmann and Metzdorf scale in relation to surgical options in the treatment of 194 follow-up patients

Group	Group A	Group B	Group C	Group D	Group E	Group F	Group G
Number of patients *N*(%)	31(16)	87(45)	6(3)	12(6)	9(5)	31(16)	18(9)
Grade 1	25	67	5	6	7	24	14
Grade 2	2	6	–	1	1	3	2
Grade 3	1	4	1	2	–	1	1
Grade 4	1	3	–	1	–	1	1
Grade 5	1	4	–	–	1	1	–
Others	1	3	–	2	–	1	–

**Fig. 1:**
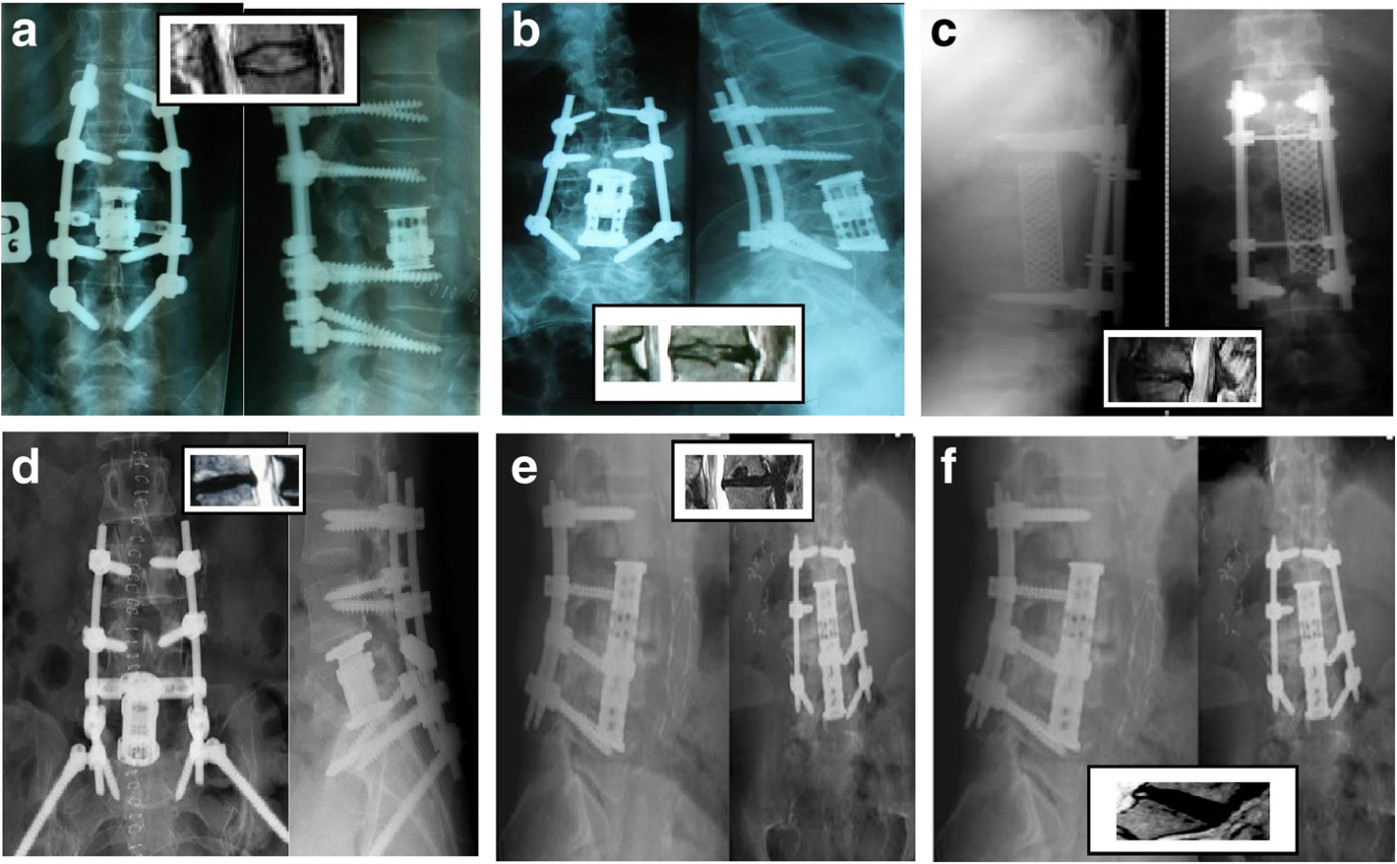
**a–f** Examples of six postoperative radiograms and MRI scans focused on different types of ASD

Figure 1(a–f) shows radiograms performed 1 year after surgery and MRI scans of adjacent segment in patients with metastatic tumor.

The study population was divided into different surgical treatment groups. No statistically significant differences in the incidence of ASD were found between these groups of patients (according to both the Pfirrmann and the Oner classifications) (Table 4).

**Table 4:** Statistical analysis of the incidence of ASD diagnosed by MRI in different groups of patients

Group	A	B	C	D	E	F	G
Number of ASD diagnosed by MRI according to the Oner, Rijt, and Ramos classifications							
*N* (%)	31 (16)	87 (45)	6 (3)	12 (6)	9 (5)	31 (16)	18 (9)
ASD *N* (%)	3 (10)	11 (13)	0 (0)	3 (25)	1 (11)	3 (10)	1 (6)
Number of ASD diagnosed by MRI according to the Pfirrmann and Metzdorf classifications							
*N* (%)	31 (16)	87 (45)	6 (3)	12 (6)	9 (5)	31 (16)	18 (9)
ASD N (%)	6 (19)	20 (23)	1 (17)	6 (50)	2 (22)	7 (23)	4 (22)

Results are presented as a number and percent

*ASD* adjacent segment disease

*p* < 0.05 for inter-group difference *χ* ^2^

Our study found significant statistical difference between the incidence of ASD cases diagnosed by the clinical examination and the incidence of ASD cases diagnosed by MRI according to the Pfirrmann et al. classification (Table 5).

**Table 5:** Statistical analysis of the incidence of ASD based on MRI and clinical examination

Detected ASD in clinical examination and MRI according to different types of radiological scale			
Scale	Oner, Rijt, and Ramos	Pfirmann and Metzdorf	Clinical signs
ASD *N* (%)	22 (11)	46 (24)*	7 (4)

Results are presented as a number and percent

*ASD* adjacent segmental disease

*p < 0.05 for inter-group difference *χ* ^2^

Tables 6, 7, 8, and 9 present the incidence of various types of ASD related to the extent of spine stabilizations.

**Table 6:** Frequency of ASD according to Oner, Rijt, and Ramos classification in patient with multilevel spine fixation

	4-level	5-level	6-level	7-level	8-level	9-level
Oner, Rijt, and Ramos						
Type 1	10	19	50	56	18	19
Type 2	1		1	3	1	1
Type 3			1	1	2	1
Type 4					1	
Type 5					1	1
Type 6	1	2		2	1	1

**Table 7:** Statistical analysis of the incidence of ASD according to stabilization extension

Oner, Rijt, and Ramos	Levels 4–6	Levels 7–9	*P* value
Types 1, 2, and 3	82	101	*ns*
Types 4, 5, and 6	3	9	<0.05

*ns* not significant

**Table 8:** Frequency of ASD according to Pfirmann and Metzdorf scale in patients with multilevel spine fixation

	4-level	5-level	6-level	7-level	8-level	9-level
Pfirmann and Metzdorf						
Grade 1	9	17	37	51	16	18
Grade 2	2	1	7	3	1	1
Grade 3		1	2	4	2	1
Grade 4		1	3	1	1	1
Grade 5		1	1	1	3	1
Others	1		2	2	1	1

**Table 9:** Statistical analysis of the incidence of ASD according to stabilization extension

Pfirmann and Metzdorf	Levels 4–6	Levels 7–9	*P* value
Grades 1 and 2	74	97	<0.05
Grades 3, 4, and 5	11	19	<0.05

We did not find any correlation between the incidence of an early developed ASD and the type of the primary tumor.

New foci of metastatic lesions in the spine were detected in 11 out of 46 patients with radiological signs of ASD. No features of spinal destabilization and local recurrence of the tumor were noted, nor were pathological fractures within the adjacent vertebrae. Ten patients were diagnosed with distortion of the sagital axis of the spine which presented as lumbar lordosis flattening or worsened thoracic kyphosis.

## Discussion

The available literature lacks data on the incidence of ASD in patients surgically treated due to metastases to the spine. Further research concerning this subject may provide valuable information on the etiological factors of the disease and improve treatment outcomes. Surgical treatment for spinal tumors is characterized by numerous differences compared with surgeries due to injuries or pathological changes. It is generally accepted to avoid bone grafting. The objective is to achieve a primary efficient stabilization, effective to the end of patient’s life. The principle is to stabilize long sections of the spine. Bone losses are filled with implants—vertebral prostheses or bone cement [14–21].

Studies on the etiology, diagnostics, and treatment of adjacent segmental disease are of vital importance because of a steady growth in spinal surgeries volumes. They are of particular relevance to young people surgically treated for disc disease, spine defects, or injuries. Studies involving patients operated on for oncological reasons in whom long sections of the spine are stabilized afford the opportunity to broaden the knowledge of the disease with additional aspects being evaluated [1–5, 7].

Harrop estimates the current state of knowledge of the factors responsible for the etiology of the disease at 35%. Among these factors, he mentions the following: reduced mobility of segments, changes in load, changes in compressibility, and pressure within a disc [5].

Lopez and Espina perceive increasing stress on the discs and endplates adjacent to the operated spinal segment as the main cause of the disease, which is confirmed by Eck [7].

Sears and Sergides et al. indicated decreased risk of ASD after spinal surgery without instrumentation. Prevalence of ASD after one-level fixation was 1.7% and 5% after three-level fixation. Laminectomy caused a 2.4 fold increase in ASD [22].

Lee et al. indicated that patients over 60 years old had a 2.5 fold increase in ASD [23].

Seevedra-Pozo et al. presented correlation between ADS and number of stabilized levels [24].

Radcliff i Kepler et al. based on MRI scans reported that the risk of ASD is 2–3% per year [25].

The early development of ASD (before 1 year) after surgery was reported by Dynesys et al and Etebar et al. They compare ASD frequency after posterior stabilization with elastic and stiff rod. In their study, MRI scans revealed ASD 9 months after surgery. Similarly Masevnin et al. (after evaluation of 120 patients) found ASD in 10 cases after two-level stabilization and 19 cases after 360 degree stabilization [26].

According to Park et al. study, the incidence of ASD increased with time from surgery. After posterior stabilization due to spondylolisthesis ASD was diagnosed in 5.2% after 36 months and among 100% after 369 months [27].

The MRI image of load-induced changes is typical. The types of degenerative changes have been demonstrated by Pfirmann and Metzdoff. They have singled out five types of the visible on MRI changes resulting from a natural progression of disc degeneration [28].

Oner, Rijt, and Ramos have differentiated between the six types of degenerative changes visible in the sagital MRI projection: type 1—normal, type 2—black disc in T2 signal, Type 3—Schmorl-type, type 4 - anterior collapse, type 5—central herniation, and type 6—degenerated disc [8].

Boden points out that 57% of patients with symptoms of ASD present no clinical signs of the disease [5].

Levin and Hale’s studies have revealed the seen on MRI radiological signs of adjacent segmental disease of the lumbar spine in 70% of cases, while clinical signs in only 36% of patients after spinal stabilization [7].

Our study has demonstrated a high incidence of ASD 1 year after spine surgeries due to cancer metastases. The most prevalent types of changes were Oner types II, III, and IV and Pfirmann types II and III. It should be noted that the criteria proposed by the authors do not overlap, and some degeneration types of Oner do not equal the types of Pfirmann. No statistically significant differences in the incidence of ASD cases were found between the study groups. The incidence of ASD cases diagnosed according to the criteria by Pfirrmann et al. (46–24%) was noticeably higher (statistically significant) than the incidence of ASD cases diagnosed according to the clinical criteria (7–3.6%). The number of clinically diagnosed ASD cases, accounting for merely 3.6% of the total study population (7/194), was significantly lower and constituted 31% of the cases diagnosed according to the Onner system and 15 of the cases diagnosed according to the Pfirrmann system. The incidence of early ASD after seven to nine levels spine stabilizations was significantly higher than after four to six levels. Our study showed higher rate of ASD 1 year after multilevel spine stabilizations than other studies.

## Conclusions

1. The prevalence of early ASD after multilevel spine stabilizations in patients with metastatic tumors is significantly higher than after surgical treatment with different underlying condition.
2. Oner types II, III, and IV and Pfirmann types II and III were most prevalent.
3. The number of ASD cases diagnosed by MRI according to the Pfirrmann classification differs from the number of ASD cases diagnosed by the clinical examination.
4. No statistically significant differences in the incidence of ASD cases between the surgical treatment groups were found.

## Abbreviation

ASD: Adjacent segmental disease
